# List of Reviewers 2023

**DOI:** 10.1192/bjo.2024.6

**Published:** 2024-04-22

On behalf of the editorial board, the Editor-in-Chief would like to thank the following for their contribution as peer reviewers in the period from January 2023 – December 2023. Their anonymous work for the journal forms the foundation to its success, and is highly appreciated.

- Abajobir, Amanuel
- Abas, Melanie
- Abdul Rashid, Aneesa
- Abe, Anne
- Adamou, Marios
- Adamson, Simon
- Adiukwu, Frances
- Admon, Roee
- Agarwal, Vivek
- Ahmed, Nilu
- Akellot, Josephine
- Alameda, Luis
- Ali, Afia
- Allison, Laura
- Al-Taiar, Hasanen
- Amering, Michaela
- Amiola, Ayomipo
- An, Zhen
- Anderson, Kelly
- Anderson, Kirstie
- Anderson, Sharon
- Andrade, Andre
- Aniwattanapong, Daruj
- Anthopolos, Rebecca
- Antypa, Niki
- Appelbaum, Paul
- Arafat, S.M. Yasir
- Araujo, Luis
- Arbour, Simone
- Archer, Charlotte
- Argyelan, Miklos
- Arnone, Danilo
- Arroyo, Bill
- Ashworth, Mark
- Bachmann, Gloria
- Badura Brack, Amy
- Bailey, Julia
- Baird, Alison
- Baker, Natasha
- Bakhla, Ajay
- Bakolis, Ioannis
- Barak, Yoram
- Baroi, Bijon
- Barra, Bernardo
- Barrett, Barbara
- Barron, Diana
- Barua, Lingkan
- Batterham, Philip
- Bayes, Adam
- Beaglehole, Ben
- Beckmann, Klaus Martin
- Beghi, Massimiliano
- Bell, Caroline
- Berenguer, Carmen
- Bergman, Yoav
- Berube, Felix-Antoine
- Besag, Frank
- Bevione, Francesco
- Bhavsar, Vishal
- Bhui, Kamaldeep
- Bielawska-Batorowicz, Eleonora
- Billings, Jo
- Birk, Max V.
- Bitta, Mary
- Black, Donald
- Blackburn, Daniel
- Blackman, Graham
- Boardman, Jed
- Boden, Joseph
- Boehnke, Jan
- Bogaers, Rebecca
- Bojanić, Lana
- Borja, Tatiane
- Bornheimer, Lindsay
- Borschmann, Rohan
- Bosma, Mark
- Bosmans, Mark
- Botbol, Michel
- Bould, Helen
- Bradfield, Owen
- Braithwaite, Isobel
- Brand, Rachel M.
- Brauer, Kay
- Bravi, Beatrice
- Breilmann, Johanna
- Brice, Samuel
- Brophy, Prof. Lisa
- Bruffaerts, Ronny
- Bucci, Paola
- Bull, Claudia
- Burke, Eilish
- Bushey, Michael
- Busuttil, Walter
- Byford, Sarah
- Cannon, Mary
- Cao, Bo
- Cardos, Roxana
- Cardozo Alarcon, Andres Camilo
- Carpena, Marina
- Carter Leno, Virginia
- Carvalho, Livia
- Caston, Frederick
- Castro, Shamyr Sulyvan de
- Ceccarelli, Caterina
- Chan, Joey WY
- Chan, Paul Shing Fong
- Chander, Russell
- Chang, Chin-Kuo
- Chang, Chunhung
- Chaudhry, Imran
- Chen, Hong-Lin
- Chen, Jianhua
- Chen, Runsen
- Cheng, Yawen
- Chester, Verity
- Chevance, Astrid
- Child, Brittany
- Chourpiliadis, Charilaos
- Chowdhury, Saifur Rhman
- Chung, Seockhoon
- Ciftci, Sezgin
- Cini, Erica
- Clark, Crystal
- Clark, David
- Clarke, Robert
- Cleare, Seonaid
- Clements, Caroline
- Cleverley, Kristin
- Cliffe, Bethany
- Cohen, Pollyanna
- Coid, Jeremy
- Comasco, Erika
- Connell, Catriona
- Conwell, Yeates
- Cookson, John
- Cooney, Emily
- Cornwall, Peter
- Cortese, Samuele
- Cote, Sarah
- Courtenay, Ken
- Crawford, Mike
- Crea, Thomas
- Crepaz-keay, David
- Crouse, Jacob
- Cubała, Wiesław
- Cullen, Prof. Kathryn R.
- Curtis, David
- Czepiel, Dianna
- Dale, Oliver
- D'Aprano, Fiore
- Daubney, Michael
- Dauvermann, Maria
- Dazzan, Paola
- De Panfilis, Chiara
- Desarkar, Pushpal
- Deuschle, Michael
- Dickens, Geoffrey L.
- Dizdarevic, Sabina
- Doherty, Anne M.
- Dom, Geert
- Donohoe, Gary
- Dorahy, Martin
- Douglas, Katie
- Dregan, Alex
- Drosos, Konstantinos
- Du, Xiangdong
- Duan, Jubao
- Duffy, Richard
- Dufour, Mathieu
- Duncan, Charlie
- Eady, Nicole
- Edbrooke-Childs, Julian
- Eddy, Clare M.
- Ede, Georgia
- Eisma, Maarten
- Elahi, Anam
- Enlow, Paul
- Ernst Nielsen, Rene
- Escobar, Javier
- Espinoza, Randall
- Fabbri, Chiara
- Fan, Amy Z.
- Fassbender, Klaus
- Favril, Louis
- Fear, Nicola
- Feeny, Norah
- Feldman, I.
- Fellinger, Matthäus
- Feng, Yan
- Fernandez-Egea, Emilio
- Ferraris, Augusto
- Finucane, Anne
- Firdosi, Mudasir
- Fischer, Claudia
- Fisher, Jane
- Flynn, Sandra
- Foerster, Katharina
- Fong, Ted
- Forbes, David
- Ford, Tamsin
- Foulds, James
- Frangou, Sophia
- Frank, Ellen
- Franklin, Catherine
- Franklin, Gary M.
- Frankova, Iryna
- Freestone, Mark
- French, Paul
- Fristad, Mary
- Fu, Yu
- Gafoor, Rafael
- Galderisi, Silvana
- Gangadharan, Satheesh Kumar
- Gangat, Abubuker
- Gannon, Louisa
- Gaughran, Fiona
- Ge, Ruiyang
- Georgescu, Raluca
- Gerace, Adam
- Gerada, Clare
- Gibbons, Robert
- Gibbs, Vicki
- Giel, Katrin
- Giordano, Giulia
- Gjestad, Rolf
- Goldstein, Benjamin
- Goodday, Sarah
- Gooding, Piers
- Gorton, Hayley
- Gournay, Kevin
- Grainge, Matthew
- Gregg, Karen
- Griffiths, Sian
- Grigg, Jonathan
- Groothuizen, Johanna
- Gupta, Mayank
- Gupta, Nihit
- Gurung, Dristy
- Hameed, Yasir
- Hampsey, Elliot
- Hardy, Amy
- Harmer, Catherine
- Harrison, Joyce
- Hashimoto, Kenji
- Haslam, Alex
- Hastings, Richard
- Hay, Phillipa
- Hazo, Jean-Baptiste
- Heald, Adrian
- Heffernan, Edward
- Heffernan, Timothy
- Henderson, Claire
- Hernández-Calle, Daniel
- Hesse, Morten
- Heumann, Eileen
- Hicks, Madelyn Hsiao-Rei
- Hicks, Paul
- Highfield, Julie
- Hill, Andrew
- Hillmer, Alannah
- Hilton, Shannon
- Ho, Roger CM
- Hoell, Andreas
- Holmes, Dawn
- Hong, You-Juan
- Hopwood, Malcolm
- Hosozawa, Mariko
- Hotopf, Matthew
- Hou, Kaijian
- Howard, Louise
- Huang, Yu-Jui
- Hull, Laura
- Humphreys, Keith
- Humpston, Clara
- Hunter, Myra
- Hunter, Rachael
- Huque, Sarah
- Husain, Mina
- Husain, Muhammad
- Inder, Maree
- Ioannidis, Konstantinos
- Isaacs, Jeremy
- Islam, Farhadul
- Janson, Staffan
- Jayakody, Dona
- Jayawickreme, Eranda
- Jefsen, Oskar
- Jelen, Luke
- Jones Nielsen, Jessica
- Jones, Brett
- Jongenelis, Michelle
- Jung, Sun Jae
- Juruena, Mario
- Kaess, Michael
- Kagee, Ashraf
- Kaiser, Stefan
- Kaiyo Utete, Malinda
- Kalfas, Michail
- Kanaan, Richard
- Kaneko, Hitoshi
- Kanneganti, Abhiram
- Kaputu-Kalala-Malu, Célestin
- Karmakar, Shubhangi
- Karmakar, Soumen
- Kaser, Muzaffer
- Kasuga, Kensaku
- Katona, Cornelius
- Kaufman, Kenneth
- Keane, Brian
- Keeley, Jared
- Kemp, Philip
- Keown, Patrick
- Kerr-Gaffney, Jess
- Keyworth, Chris
- Kilic, Mahmut
- Kim, Junpyo
- Kim, Sun Mi
- Kindler, Jochen
- King, Gabriella
- King, Toni
- Kirkpatrick, Brian
- Kisely, Steve
- Knapp, Martin
- Knowles, Gemma
- Kobach, Anke
- Kobulsky, Julia M.
- Koike, Shinsuke
- Kolli, Venkat
- Kolshus, E.
- Konstantakopoulos, George
- Koops, E
- Kopp, Svenny
- Koren, Dan
- Koutsouleris, Nikolaos
- Kraepelien, Martin
- Kühner, Christine
- Kundu, Satyajit
- Kurdyak, Paul
- Lacey, Rebecca
- Ladergard, Kristie
- Lai, Daniel
- Laing, Ian
- Lam, Austin
- Lam, Janet
- Lang, Yanda
- Lappin, Julia
- Large, Matthew
- Laugharne, Richard
- Launders, Naomi
- Lavdas, Michail
- Lawrence, Peter
- Lawrie, Stephen
- Lazzarino, Antonio
- Lazzaro, Carlo
- Lee, Allen TC
- Lee, Jimmy
- Lee, Kounseok
- Lee, Rachel
- Lee, wanhyung
- Lee, William
- Lenferink, Lonneke
- León-Ortiz, Pablo
- Lepping, Peter
- Leucht, Stefan
- Lewis, Anne
- Li, Jialiang
- Li, Jing
- Li, Kaiyun
- Li, Su-Xia
- Lidlington, Emma
- Lin, Chung-Ying
- Lin, Rongmao
- Liu, Jialiang
- Liu, Chaomeng
- Lotzin, Annett
- Lovik, Anikó
- Lü, Wenqi
- Lucassen, Mathijs
- Luciano, Mario
- Lukic, Marko
- Lunsky, Yona
- Luo, Qinghua
- Lynch, Sean
- Lyu, Bei
- Mac Giollabhui, Naoise
- Maccallum, Fiona
- Mackenzie, Jay-Marie
- Malhi, Gin
- Mane, Anna
- Mann, John
- Manning, Victoria
- Markevych, Iana
- Markham, Sarah
- Markkula, Niina
- Markt, Afra
- Marshall, Nicola
- Martino, Gabriella
- Martins, Daniel
- Martorana, Alessandro
- Maruyama, Jessica
- Maselko, Joanna
- Mason, Oliver
- Matsunaga, Asami
- Mattison, Richard
- Max, Sebastian
- Mazibuko, Sindisiwe
- McDaid, David
- McGee, Jocelyn S.
- McGorry, Patrick
- McGuinness, Elizabeth
- McIntosh, Virginia
- McIver, Theresa
- McLoughlin, Declan
- McManus, Sally
- McSherry, Bernadette
- Meader, Nick
- Medhurst, David
- Mehta, Mitul
- Mekonnen, Habtamu
- Melchior, Maria
- Meltzer, Herbert
- Melvin, Clare
- Merritt, Carrington C.
- Miller, Stephen
- Milte, Dr Rachel
- Milton, Alyssa
- Mitchell, Kirstin
- Mitrou, Francis
- Molodynski, Andrew
- Monga, Suneeta
- Monk, Nathan
- Morbelli, Silvia Daniela
- Morentin, Benito
- Morton, Emma
- Moseley, Rachel
- Moulton, Vanessa
- Mucci, Armida
- Muehlhan, Markus
- Mueller, Marie
- Mühlmann, Marlene
- Mukherjee, Soumyadeep
- Mula, Marco
- Mulder, Roger
- Münker, Lina
- Muñoz-Navarro, Roger
- Munshi, Tariq
- Murphy, Glynis
- Murray, Esther
- Murtagh, Fliss E. M.
- Musavi, Zeinab
- Mutz, Julian
- Nasser, Zeina
- Natesan Batley, Prathiba
- Nemeroff, Charles B.
- Newbury, Joanne
- Newlove-Delgado, Tamsin
- Nguyen, Minh-Hoang
- Nickerson, Angela
- Nicoletti, Ferdinando
- Niedzwiedz, Claire
- Nielssen, Olav
- Nikoloski, Zlatko
- Nilsson, Thomas
- Noblett, Joanne
- Ntontis, Evangelos
- Nunes, Abraham
- Nunez, John-Jose
- O'Connor, Maja
- Oduola, Sherifat
- Oexle, Nathalie
- Ohara, Tadahisa
- Okasha, Tarek
- Okpaku, Sam
- Olashore, Anthony
- Olié, Emilie
- Oliveira, Paula
- Oliver, Dominic
- Oliver, Lindsay
- Oltean Cardos, Roxana
- Opitz, tine
- O'Reilly, Dermot
- Ortega, Francisco
- Ortiz, Abigail
- Osvath, Peter
- Ouali, Uta
- Ougrin, Dennis
- Ouma, Simple
- Owen, Gareth
- Pala, Kayıhan
- Palinkas, Lawrence
- Palmer, Christopher
- Paloyelis, Yannis
- Pang, Nicholas Tze Ping
- Panzavolta, Andrea
- Paret, Christian
- Pariwatcharakul, Pornjira
- Park, A-La
- Parker, Gordon
- Partonen, Timo
- Payne, Leanne
- Peltzer, Karl
- Penney, Stephanie
- Perakyla, Anssi
- Perez, David
- Petkova, Eva
- Petti, Theodore
- Pezzella, Pasquale
- Phelps, James
- Phillips, Thomas
- Philpot, Ursula
- Pick, Susannah
- Pike, Alexandra
- Pingani, Luca
- Pinto da Costa, Mariana
- Pipitone, Nathan
- Pitanupong, Jarurin
- Pitman, Alexandra
- Pitron, Victor
- Pizzagalli, Diego
- Plana-Ripoll, Oleguer
- Pokhilenko, Irina
- Polanczyk, Guilherme
- Pollard, Martha
- Pompili, Maurizio
- Popovic, David
- Porter, Richard
- Pourriyahi, Homa
- Price, Annabel
- Prigerson, Holly
- Pulopulos, Matias M.
- Purcell, Julian
- Putranto, Rudi
- Quadros, Wesley
- Quinlivan, Leah
- Rabasco, Ana
- Rabei, Samah
- Rachul, Christen
- Ramaboa, Kutlwano
- Ramsay, Russell
- Randles, Rebecca
- Rawther, Aquil
- Rebillat, Anne-Sophie
- Regnard, Claud
- Reilly, Joe
- Reilly, Thomas
- Reinders, Antje
- Reynolds, Charles
- Reynolds, Edward
- Rezel-Potts, Emma
- Riedel-Heller, Steffi
- Rinaldi, Miles
- Ring, David
- Rivart, Pauline
- Robinson, Tomos
- Rockett, Ian R. H.
- Rosen, Stuart
- Rowe, Sarah
- Roy, Ashok
- Roy, Nitai
- Rucci, Paola
- Rucker, James
- Ruengorn, Chianchong
- Rugkasa, Jorun
- Rush, Augustus
- Ryan, Brendan
- Ryu, Wonjung
- Sackeim, Harold
- Sahota, Gurvinder
- Sakata, Masatsugu
- Salisbury, Tatiana
- Salpekar, Jay
- Samsel, Chase
- Sancho-Domingo, Clara
- Sander, Lasse
- Sangraula, Manaswi
- Sanwald, Simon
- Saranathan, Manojkumar
- Sarkar, Sameer
- Saunders, Rob
- Schaffer, Ayal
- Schetz, Daria
- Schleicher, Daniel
- Schomerus, Georg
- Schulze, Thomas
- Seeman, Mary V.
- Selby, Edward
- Serafini, Gianluca
- Serrell, Jerrie
- Shacham, Maayan
- Shahbaz, Shumaila
- Shahid, Syed Kashif
- Shaji, Aparna
- Sham, Pak Chung
- Shankar, Rohit
- Shapira, Stav
- Sharwood, Lisa
- Shenoi, Asha N.
- Shepherd, Andrew
- Shevlin, Mark
- Shim, Ruth
- Shin, Cheolmin
- Shrira, Amit
- Silva, Ed
- Simms, Amos
- Simpson, Alan
- Singh, Bruce
- Siwek, Marcin
- Skuse, David
- Smith, Shubulade
- Smyrnis, Nikolaos
- Solmi, Francesca
- Somasundaram, Daya
- Son, Dahee
- Soneson, Emma
- Soriano-Mas, Carles
- Soteriades, Elpidophoros
- Sousa, Soraia
- Spangenberg, Lena
- Sparling, Thalia
- Speerforck, Sven
- Speyer, Helene
- Stangeland, Helle
- Stavert, Jill
- Steare, Thomas
- Steele, Rachel
- Steinert, Tilman
- Stewart, Robert
- Stingl, Julia
- Strawbridge, Rebecca
- Strouza, Aikaterini
- Stuart, Simon
- Stummer, Harald
- Sucher, Catherine
- Sulaiman-Hill, Ruqayya
- Suzuki, Takefumi
- Takei, Nori
- Tamam, Lut
- Tan, Diana
- Tan, Jacinta
- Tarokh, Leila
- Tarricone, Ilaria
- Tay, Alvin Kuowei
- Taylor, David
- Taylor, Hugh
- Tchanturia, Kate
- Tennant, Matthew
- Tetkovic, Irena
- Teunis, Teun
- Thach, Thuan
- Tham, Su-Gwan
- Thiago, Roza
- Tint, Ami
- Tomczyk, Samuel
- Tondo, Leonardo
- Tong, Yongsheng
- Torous, John
- Tournier, Marie
- Tracy, Derek
- Trautmann, Sebastian
- Tromans, Samuel
- Trompeter, Nora
- Tsamakis, Konstantinos
- Tsapara, Angeliki
- Tsiachristas, Apostolos
- Turner, James
- Tyrer, Freya
- Tyrer, Peter
- van Amsteram, Jan
- Van Elburg, Annemarie
- Varese, Filippo
- Ventevogel, Pieter
- Vigod, Simone
- Villarreal, Beltran
- Vogt, Kathy
- Voniati, Louiza
- Waller, Glenn
- Wang, Jianbing
- Wearne, Deborah
- Weissman, Myrna
- Weller, Penelope
- Wells, Imogen
- Werner, Perla
- Wessely, Simon
- Westbury, Juanita
- Westlund Schreiner, Mindy
- Wheeler, Nicola
- Whelan, Paul
- Wicke, Felix
- Willey, Suzanne
- Williams, Richard
- Wilson, Claire
- Wilson, Jack
- Wise, Toby
- Wisse, Laura Em
- Witthöft, Michael
- Wolverson, Emma
- Woodrow, Amanda
- Worhunsky, Patrick D.
- Worthen, Miranda
- Wright, Dragana
- Wu, Jingwei
- Wu, Shaolong
- Wykes, Til
- Xing, Xiu-Xia
- Xu, Shu-Hui
- Yamaguchi, Sosei
- Yang, Min
- Yanos, Philip
- Yates, Susan
- Yon, Dong Keon
- Yonemoto, Naohiro
- Young, Allan
- Zagożdżon, Paweł
- Zahn, Roland
- Zalewska, Anna M.
- Zangani, Caroline
- Zellers, Stephanie
- Zhang, Hanting (Hunter)
- Zhang, Jihui
- Zhang, Nanhua
- Zhang, Shuo
- Zhukovsky, Peter

